# Blood–brain barrier disruption and ventricular enlargement are the earliest neuropathological changes in rats with repeated sub-concussive impacts over 2 weeks

**DOI:** 10.1038/s41598-021-88854-9

**Published:** 2021-04-29

**Authors:** Bailey Hiles-Murison, Andrew P. Lavender, Mark J. Hackett, Joshua J. Armstrong, Michael Nesbit, Samuel Rawlings, Terrence McGonigle, Andrew Warnock, Virginie Lam, John C. L. Mamo, Melinda Fitzgerald, Ryu Takechi

**Affiliations:** 1 Curtin Health Innovation Research Institute, Curtin University, Bentley, WA Australia; 2 Curtin Medical School, Faculty of Health Sciences, Curtin University, Bentley, WA Australia; 3 School of Science, Psychology and Sport, Federation University Australia, Mount Helen, VIC Australia; 4 School of Allied Health, Faculty of Health Sciences, Curtin University, Bentley, WA Australia; 5 School of Molecular and Life Sciences, Faculty of Science and Engineering, Curtin University, Bentley, WA Australia; 6 School of Population Health, Faculty of Health Sciences, Curtin University, Bentley, WA Australia; 7 Perron Institute for Neurological and Translational Science, Ralph and Patricia Sarich Neuroscience Research Institute, Nedlands, WA Australia

**Keywords:** Blood-brain barrier, Neurological disorders

## Abstract

Repeated sub-concussive impact (e.g*.* soccer ball heading), a significantly lighter form of mild traumatic brain injury, is increasingly suggested to cumulatively alter brain structure and compromise neurobehavioural function in the long-term. However, the underlying mechanisms whereby repeated long-term sub-concussion induces cerebral structural and neurobehavioural changes are currently unknown. Here, we utilised an established rat model to investigate the effects of repeated sub-concussion on size of lateral ventricles, cerebrovascular blood–brain barrier (BBB) integrity, neuroinflammation, oxidative stress, and biochemical distribution. Following repeated sub-concussion 3 days per week for 2 weeks, the rats showed significantly enlarged lateral ventricles compared with the rats receiving sham-only procedure. The sub-concussive rats also presented significant BBB dysfunction in the cerebral cortex and hippocampal formation, whilst neuromotor function assessed by beamwalk and rotarod tests were comparable to the sham rats. Immunofluorescent and spectroscopic microscopy analyses revealed no significant changes in neuroinflammation, oxidative stress, lipid distribution or protein aggregation, within the hippocampus and cortex. These data collectively indicate that repeated sub-concussion for 2 weeks induce significant ventriculomegaly and BBB disruption, preceding neuromotor deficits.

## Introduction

Whilst concussion or mild traumatic brain injury (mTBI) is commonly indicated by loss of consciousness, transient cognitive dysfunction and memory loss, as a product of rapid-onset biomechanical trauma and associated cellular insult^1–3^, sub-concussion is a much lighter form of head impact that does not produce discernible clinical symptoms comparable to concussion^4^. There is markedly greater interest in the potential chronic effects of repeated sub-concussion^1^ and increasing evidence that repetitive sub-concussion can cumulatively result in considerable adverse effects on brain anatomy and function^5–9^. A study by Lipton et al. noted that soccer players with increased exposures to “headings” exhibited a decrease in neurocognitive performance as well as notable alterations to white matter microstructure which can lead to complex and severe neurodegenerative diseases^10^. Whilst clinically less severe, sub-concussion may nonetheless be a risk factor for neurodegenerative processes. However, the mechanisms by which repeated sub-concussion putatively compromises the central nervous system is presently unknown^5^.

It is increasingly recognised that the integrity of the cerebrovascular blood–brain barrier (BBB) plays a critical role in maintaining healthy neuronal function and neurobehavioural performance. Indeed, breach of the BBB results in cerebral extravasation of blood–borne potentially neuroinflammatory molecules and immune cells, astrogliosis, heightened mitochondrial activity, oxidative stress, neurovascular inflammation and neurodegeneration^11^. The balanced metabolic connections between glia and neurons are essential to preserving a healthy central nervous system as well as neuronal physiology and function^12,13^. As such, disruptions to metabolism and holistic brain biochemistry in the brain could play a role in the development of certain neurodegenerative diseases. Literature indicates most neurodegenerative diseases have an element of disturbed metabolism and biochemical dyshomeostasis, so while they are not necessarily the primary cause of disease, this change in regulation may increase the risk of disease^14^. Indeed, dysregulation of metals, lipids and protein aggregation in the brain have been a focal point in the study of a plethora of neurodegenerative diseases over the past decade; including Alzheimer’s disease^15^. A study conducted by Fimognari et al. showed evidence of increased lipid saturation and zinc deficiency in a specific sub-region of the hippocampal formation in a murine model of accelerated ageing and neurocognitive deficits^16,17^. Whilst the changes in cortical and hippocampal biochemical homeostasis are of interest, they have never been studied in relation to repeated sub-concussion. Thus, the investigation of dysregulated brain biochemistry may provide informative mechanistic insights on the development of neurobehavioural disorders following long-term repeated sub-concussive impacts.

Recently, we established a rat model of repeated sub-concussion and reported that sub-concussive impacts repeated for 12 weeks induced significant neuromotor deficits, indicated by increased foot errors on the beamwalk test^18^. However, neuromotor dysfunction was not evident following repeated sub-concussion after a shorter period of 2 weeks. In the pilot report however, ex vivo neuropathological analyses were not conducted and hence, the underlying mechanisms that preceded the neurobehavioural deficits in long-term repeated sub-concussion rats remain elusive. Therefore, in the present study, we investigate the neuropathological changes that preceded the neuromotor deficits in the brain and plasma samples of the rats that received repeated sub-concussive impact for 2-weeks, as described in our previous study^18^.

## Results

### Repeated sub-concussion did not cause clinical adverse events

During the 2-week repeated sub-concussion/sham procedure, the Sham and sub-concussion (SC) rats were closely monitored for any adverse effects and clinical signs including pain, seizure, lethargy and weight loss. The sub-concussive impacts were well-tolerated and no adverse events were observed. There was no significant differences in the weight gain between the rats receiving repeated sub-concussion and sham procedure (Fig. S1). The recovery time from the isoflurane anaesthesia in the SC group rats did not significantly differ from the Sham group rats (Fig. S1). Following the recovery, the behaviour and activity of rats in the SC group were comparable to the Sham rats and no rats exhibited sign of pain, skull injury or seizure.

### Repeated sub-concussion did not induce detectable neuromotor dysfunction

**Figure 1:**
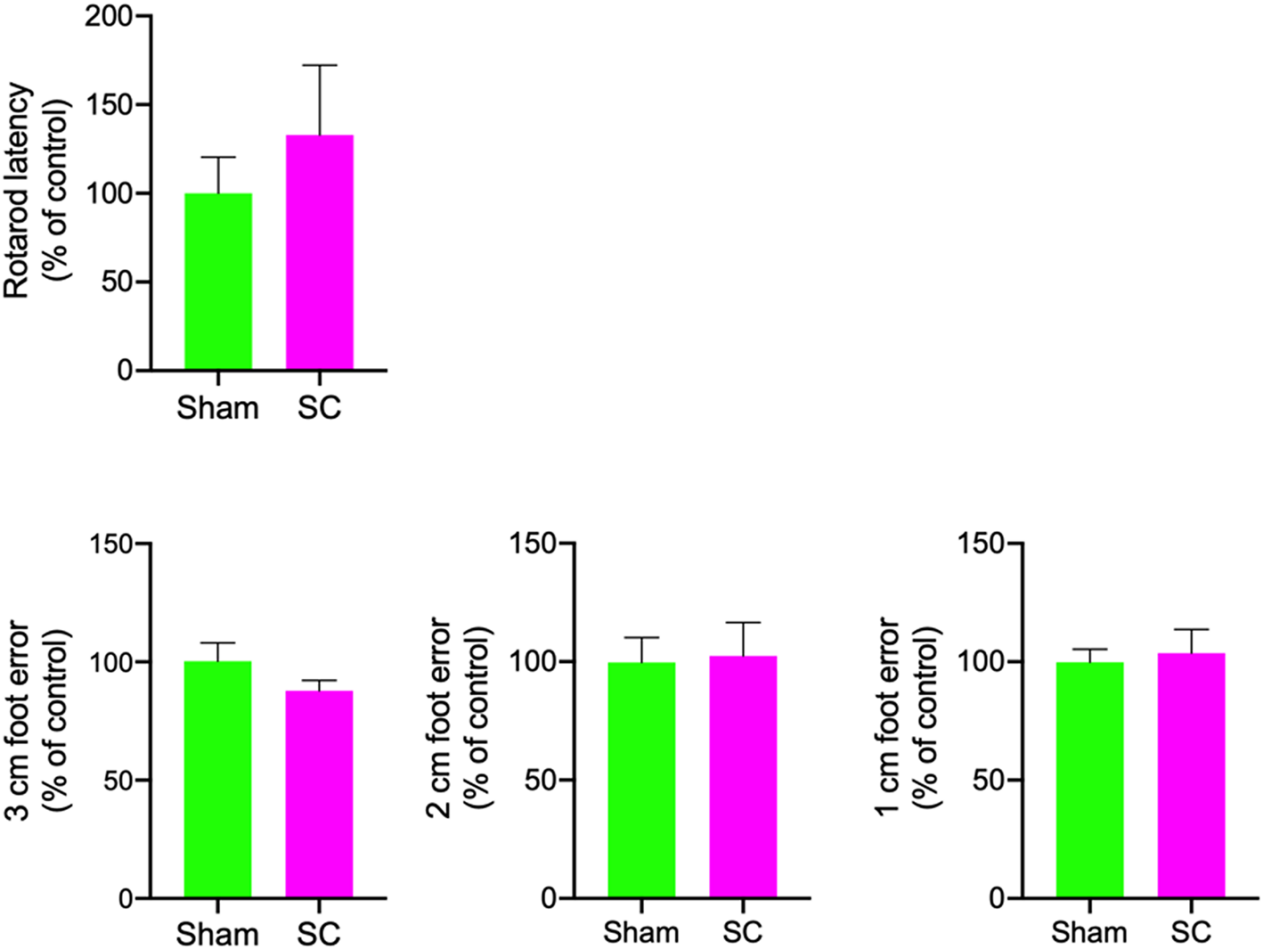
Neuromotor performance. Neuromotor performance in rats receiving repeated sub-concussion (SC) or sham procedure for 2 weeks were assessed with rotarod and beamwalk tests using 3, 2, and 1 cm. The latency of rats stayed on the rotating rod of rotarod test is presented as percent of the mean of Sham control group (mean ± SEM). The number of foot errors on 3, 2, and 1 cm beamwalk are also shown as percent of the Sham control group. Statistical significance was assessed with 2 tailed t-test (*p* < 0.05, n = 8). (adapted from Lavender et al.^18^).

Neuromotor performance was assessed with beamwalk and rotarod tests following the completion of 2-week repeated sub-concussion or sham procedure and previously reported^18^. The rats that received repeated sub-concussive impacts for 2 weeks showed no significant difference in the rotarod latency to remain on the rotating rod, compared to the rats that underwent only sham procedure (Fig. 1). The beamwalk foot error on 3 cm wide beam in SC group rats was comparable to the foot error of Sham group rats (Fig. 1). Similarly, no significant difference was observed in the 2 cm beamwalk foot error between the rats with Sham and SC procedure. The foot error on 1 cm beamwalk was also comparable between the SC and Sham groups.

### 2-week repeated sub-concussion led to ventriculomegaly

**Figure 2:**
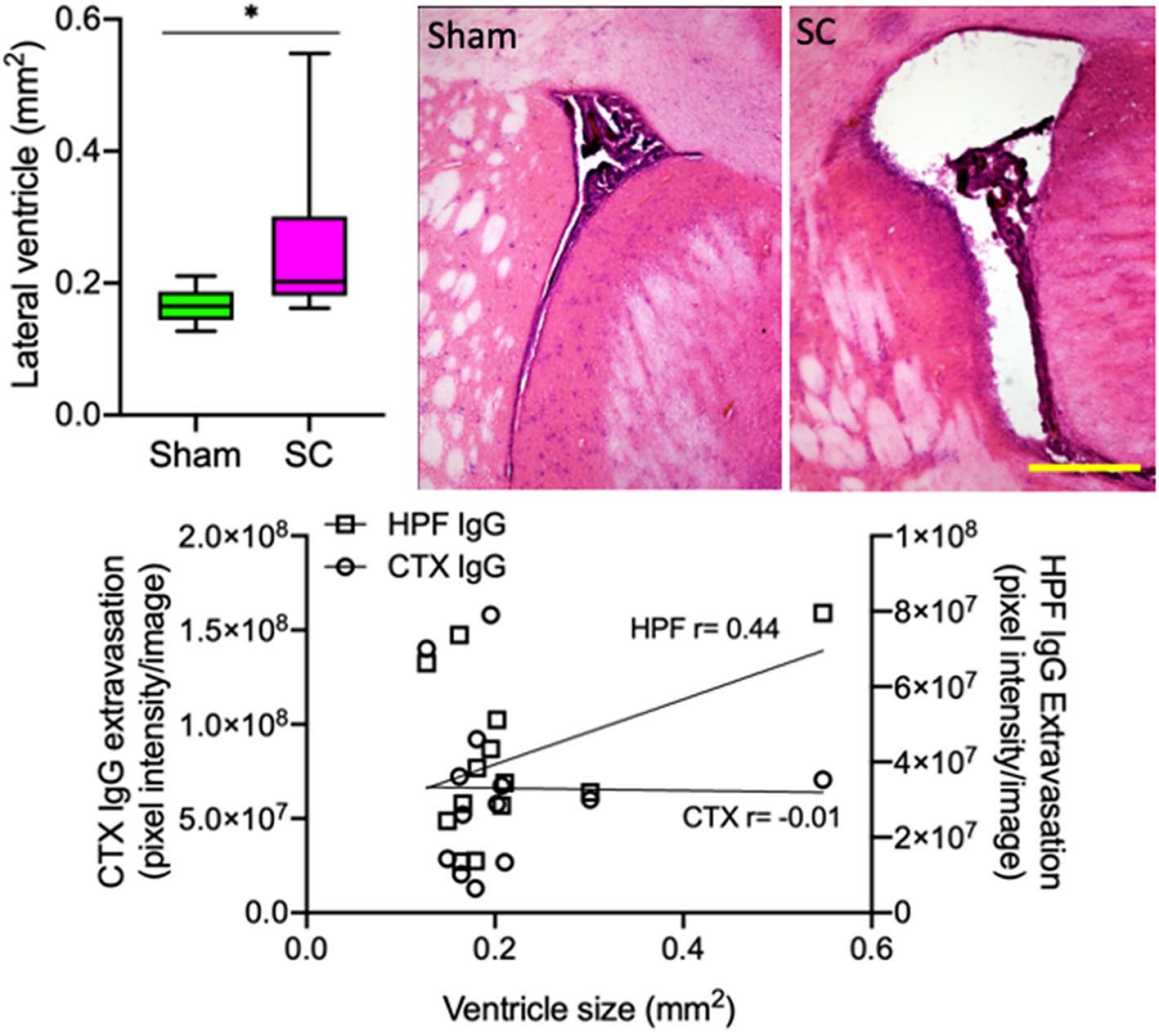
Lateral ventricular size. The lateral ventricle size was measured at Bregma 0 mm in sub-concussion rats (SC) (n = 6) and sham (n = 7) rats. The data is shown as the lateral ventricle area size as median ± 95% confidence interval and * indicates statistical significance at *p* < 0.05 assessed with Mann–Whitney test. Representative lateral ventricle microscopy images from SC and Sham rats are also presented (scale bar = 500 µm). In the lower frame, the Spearman’s correlation graph is presented to demonstrate the association between lateral ventricle size and IgG extravasation in cortex (CTX) or hippocampus (HPF).

The area of lateral ventricle at Bregma 0 mm was measured histologically in the fixed-frozen right hemisphere. The results indicated a significant enlargement of lateral ventricle in the rats receiving 2-week repeated sub-concussive impacts, in comparison to the rats receiving sham-only procedure for 2 weeks (Fig. 2). The Pearson’s correlation coefficient analysis indicated moderate association (r = 0.44) between the lateral ventricle enlargement and BBB disruption within the hippocampal formation, whereas no association (r = −0.01) was found with the BBB dysfunction in cortex.

### Significant BBB disruption was observed after repeated sub-concussion

**Figure 3:**
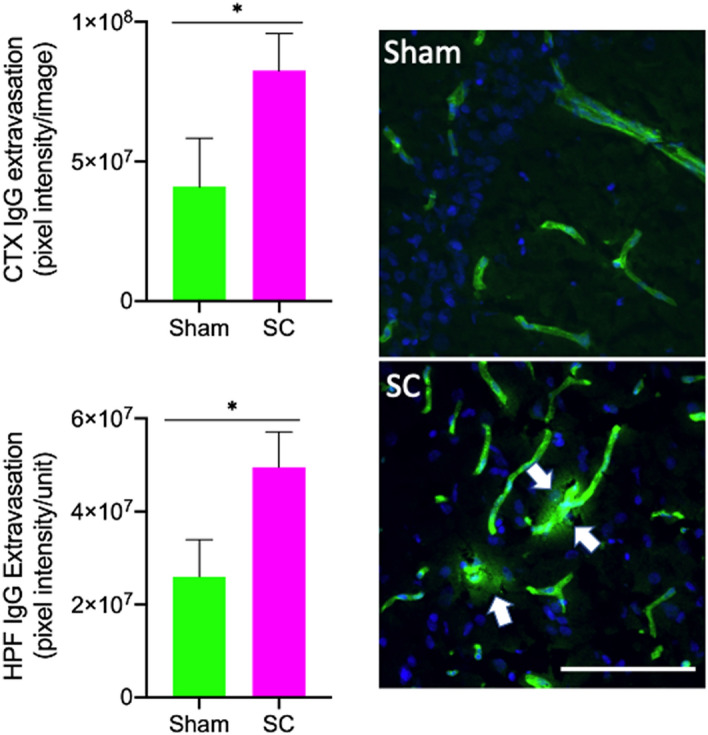
BBB permeability. The integrity of BBB was assessed with immunofluorescent microscopy measuring the parenchymal perivascular extravasation of IgG in rats that received either sham or repeated sub-concussion (SC) procedure for 2 weeks. The IgG extravasation was semi-quantitatively determined in the cortex (CTX) and hippocampal formation (HPF) regions and expressed pixel intensity per image (mean ± SEM, left frame). Representative immunomicroscopy images are presented on the right with IgG shown in green and perivascular leakage indicated with white arrows (scale bar = 100 µm). Statistical significance was determined with t-test (*p < 0.05, n = 8).

The integrity of BBB was assessed in the cortex and hippocampal formation by quantifying the parenchymal perivascular extravasation of plasma IgG. After receiving repeated sub-concussion for 2 weeks, the rats showed significantly exaggerated cortical perivascular IgG extravasation compared to the rats receiving sham procedure, indicating substantially increased BBB permeability (Fig. 3). The 2-week repeated sub-concussion resulted in substantial BBB disruption in the hippocampal region, leading to a significantly greater parenchymal IgG leakage comparing to the Sham group rats.

### Plasma cytokines

**Figure 4:**
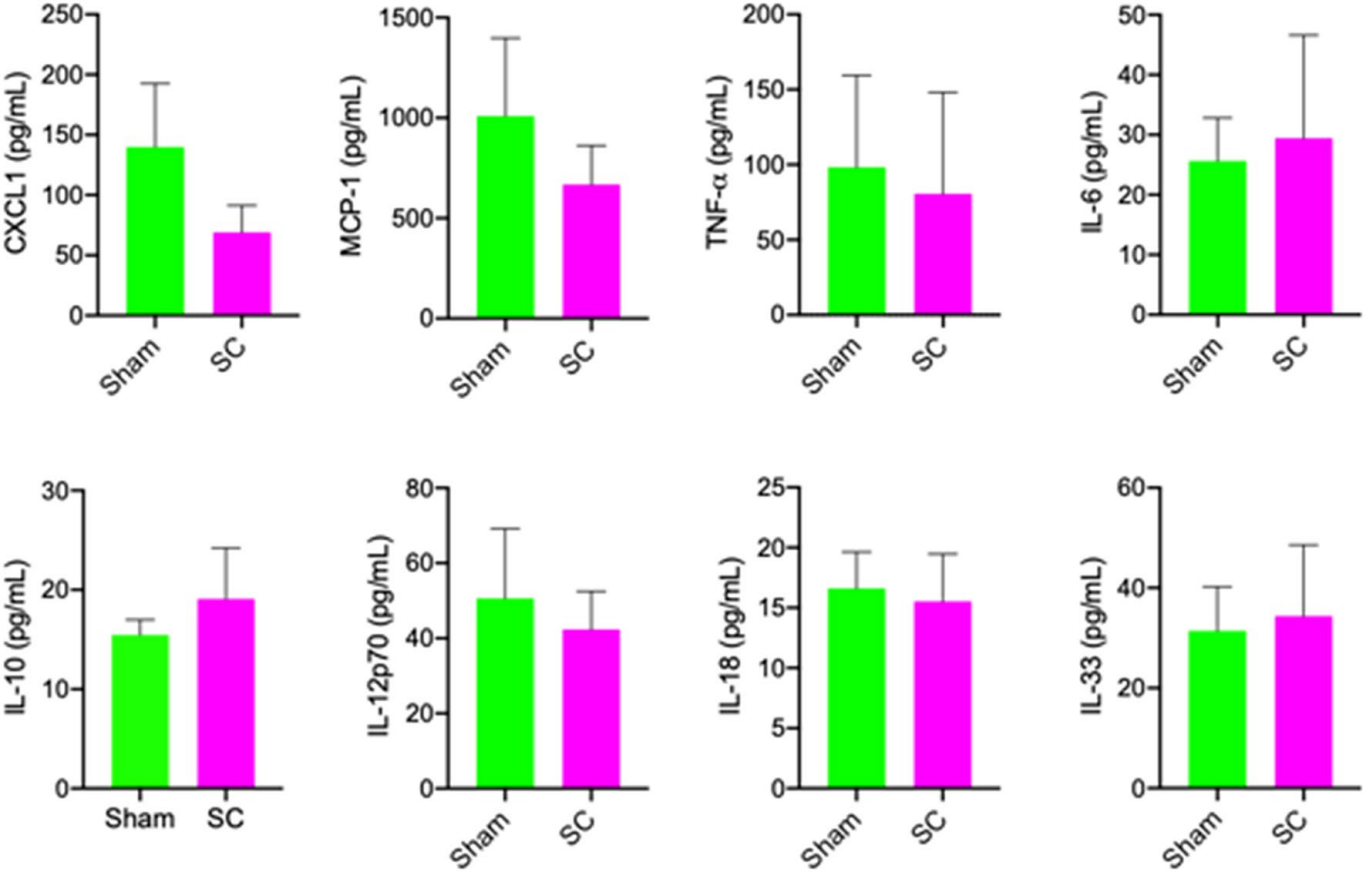
Plasma inflammatory cytokines. The plasma concentrations of pro- and anti-inflammatory cytokines were measured with flow cytometry beads array in rats receiving sub-concussion (SC) or sham procedure for 2 weeks. The data is shown as mean ± SEM. Statistical significance was assessed with unpaired t-test (*p < 0.05).

Plasma levels of pro- and anti-inflammatory chemokines and cytokines were analysed with flow cytometry beads array. In rats that received 2-week sub-concussion, the mean plasma concentration of pro-inflammatory chemokine, CXCL1 showed approximately 100% reduction compared to the Sham group rats, although it did not reach statistical significance at *p* < 0.05 (Fig. 4). A pro-inflammatory cytokine, MCP-1 was also reduced by approximately 35% in SC group rats compared to the Sham rats, although it was not statistically significant. Other pro-inflammatory cytokines including TNF-α, IL-6, IL-12p70, IL18 and IL-33, were comparable between the SC and Sham groups (Fig. 4). The plasma concentration of anti-inflammatory IL-10 was also similar in SC and Sham group rats.

### Neuroinflammation and oxidative stress were not evident in SC rat brain

**Figure 5:**
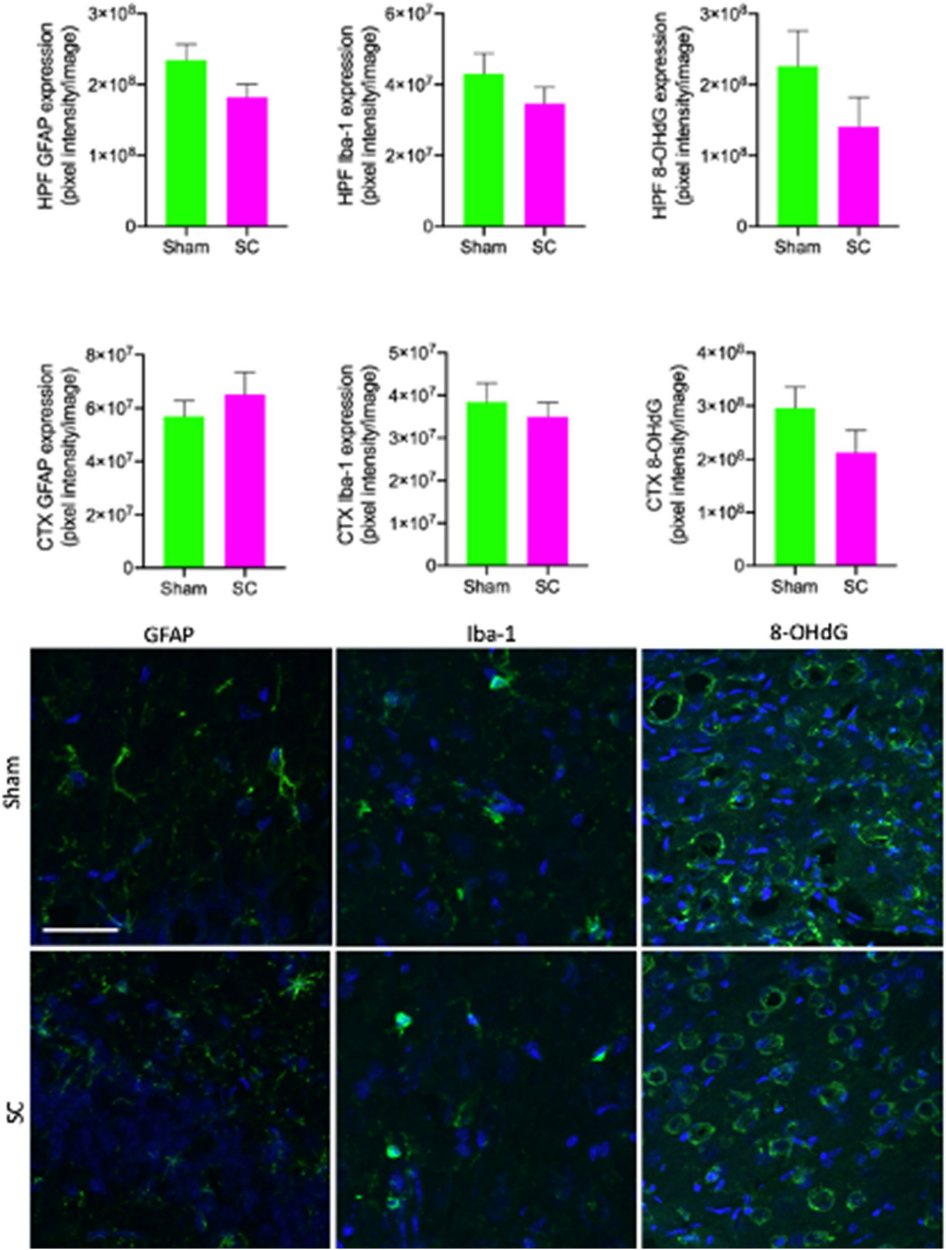
Neuroinflammation and oxidative stress in hippocampal formation and cortex. The markers of neuroinflammation (GFAP and Iba-1) and oxidative stress (8-OHdG) were semi-quantitatively measured in the hippocampal formation (HPF) and cortex (CTX) of sub-concussion rats (SC) and sham group. The data is shown as the mean pixel intensity per image with SEM. Statistical significance was assessed with two-tailed t-test at *p* < 0.05 (n = 8). Representative immunofluorescent micrographs are presented in the lower frame (scale bar = 40 µm). GFAP, Iba-1 and 8-OHdG are shown in green, while the nuclei DAPI staining is shown in blue.

Following the 2-week SC/Sham procedure, the neuroinflammation and oxidative stress markers were measured in the hippocampus and cortex with immunofluorescent microscopy. In the hippocampus of rats receiving repeated sub-concussive impacts, the immunoreactivity of GFAP was not significantly different from the rats receiving sham procedure, indicating no evidence of astrocytosis in SC rats’ hippocampus (Fig. 5). Similarly, in the hippocampus of SC rats, the Iba-1 abundance was comparable to the Sham rats, demonstrating no increase in microglial activation induced by the repeated sub-concussion. DNA oxidative stress damage in the hippocampal formation indicated with the immunoreactivity of 8-OHdG was also similar between the SC and Sham group rats.

In the cortex of the rats that underwent 2-week sub-concussion procedure, the abundance of GFAP was similar to the rats receiving sham-only procedure (Fig. 5). Similarly, the cortical levels of Iba-1 in SC group rats were comparable to the Sham group rats. The levels of oxidative stress marker, 8-OHdG, was also similar between the Sham and SC groups.

### FTIR spectroscopy revealed no significant biochemical changes in the HPF and CTX

**Figure 6:**
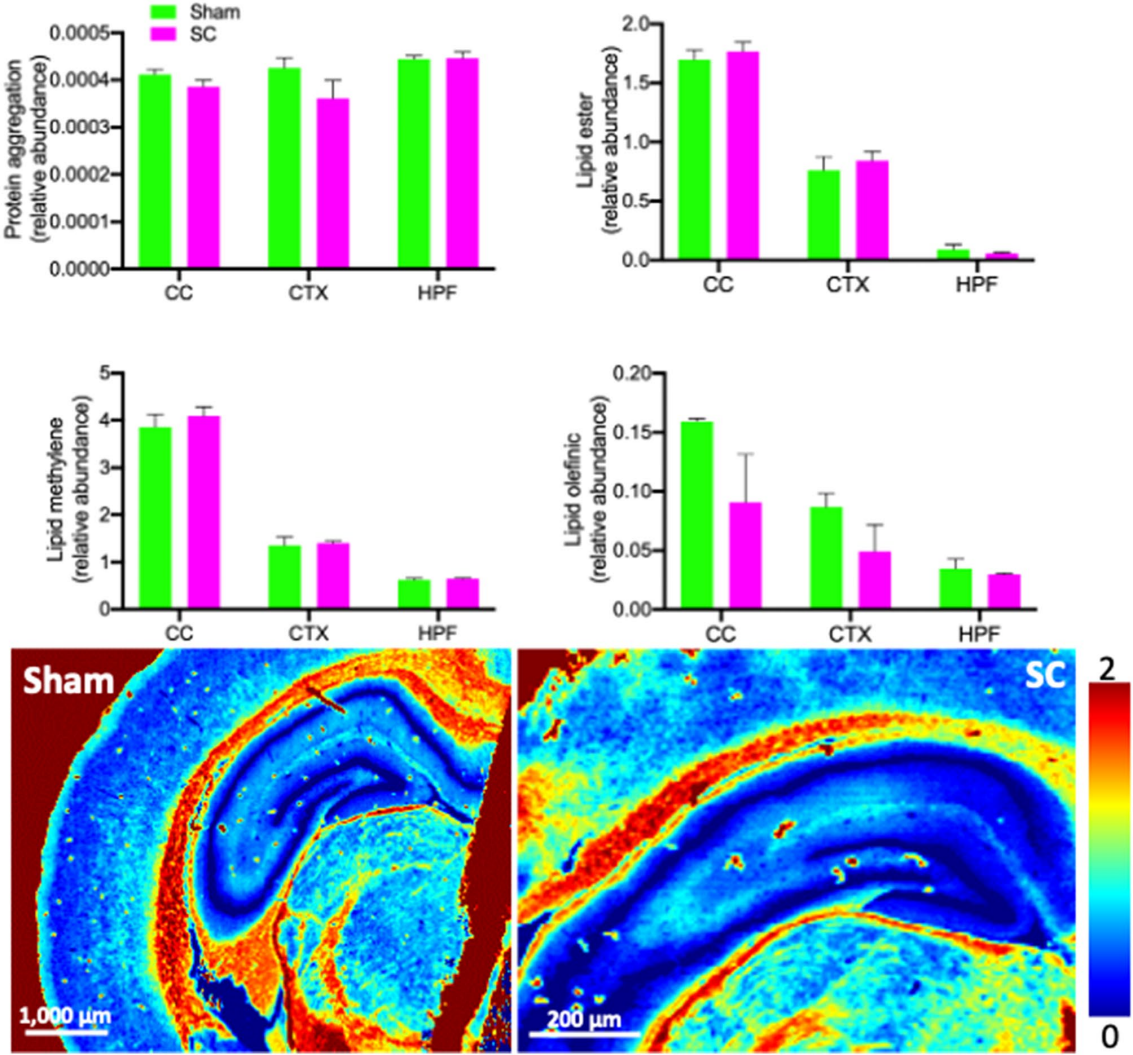
Lipid distribution and protein aggregation in the hippocampal formation and cortex. The changes in the biochemical distribution in the corpus callosum (CC), hippocampus (HPF) and cortex (CTX) of sub-concussion rats (SC) and sham group were analysed with FTIR spectroscopy. The data is shown as the mean relative abundance per area with SEM. Statistical significance was assessed with two-tailed t-test at *p* < 0.05 (n = 4). Representative spectroscopy images are presented in the lower frame.

FTIR spectroscopic imaging was performed at 25 µm spatial resolution to investigate holistic biochemical changes that may have occurred following repeated sub-concussive impacts. Protein aggregation was assessed through analysis of second-derivative intensity at 1625 cm^−1^, as increased intensity at this spectroscopic location is associated with increased relative abundance of aggregated proteins with a β-sheet secondary structure^19^. In this study, the 2-week repeated sub-concussion did not significantly alter the protein aggregation levels in the corpus callosum, cortex and hippocampal formation, compared to the sham group rats (Fig. 6).

Alterations to lipids were tested through analysis of band areas associated with lipid esters [ν(C=O), 1760–1715 cm^−1^], lipid methylene groups [ν_s_(CH_2_), 2865–2840 cm^−1^], and lipid unsaturation [ν(= C–H), 3025–3000 cm^−1^]. Semi-quantitative abundance of lipid ester, lipid methylene and lipid olefinic distribution within the corpus callosum, cortex and hippocampal neurons and representative false-colour FTIR images of lipid distribution (lipid ester), in sham and SC animals are presented in Fig. 6. The levels of lipid ester in the corpus callosum, cortex, and hippocampal formation, were not significantly different between the Sham group and SC group rats. The repeated sub-concussion did not alter the distribution of lipid methylene in the corpus callosum, cortex and hippocampus in comparison to the Sham group. The levels of lipid olefinic in the corpus callosum and cortex were reduced by 43% and 44%, respectively, in the SC rats compared to the Sham rats, although it was not statistically significant. The distribution of lipid olefinic in the hippocampal formation was comparable between SC and Sham groups.

## Discussion

An emerging body of literature suggests that repeated sub-concussive head impacts over an extended period may cumulatively induce significant structural and functional changes to the brain. However, the underlying mechanisms have not been extensively studied to date. The present study utilised for the first time, a rat model to investigate the potential underlying mechanistic pathways that precede the neurobehavioural and cerebral structural alterations induced by long-term repeated sub-concussion.

We recently established a novel rat model of repeated sub-concussion, which was aimed to replicate clinical long-term repetitive sub-concussion such as soccer heading^18^. In the study, the rats receiving repeated sub-concussive impacts for 12 weeks showed significant neuromotor deficits indicated by the increase in beam walk foot errors, whereas the rats receiving repeated sub-concussion for 2 weeks showed no signs of neuromotor dysfunction. In order to explore potential pathways involved in the sub-concussion-induced neuromotor deficits, we here utilised the rat brain and plasma specimens collected from the rats following the 2-week repeated sub-concussion impacts to investigate neuropathological changes that preceded the development of apparent neuromotor dysfunction. The SC group rats did not show any neuromotor dysfunction, clinical signs or activity/behavioural changes such as seizure following the 2-week repeated sub-concussion procedure, consistent with the clinical definition of sub-concussion^7^.

Remarkably, our histological analyses indicated significantly increased lateral ventricle area in the rats with repeated sub-concussion, indicating substantial brain structural changes occurring without any symptomatic or neurobehavioural changes. Ventriculomegaly is indeed a typical neuropathological feature of chronic traumatic encephalopathy (CTE) that is induced as a result of concussive head injury^20^. Geddes et al. reported that CTE and ventricle enlargement are an early consequence of repetitive concussive impacts in soccer players^21^. Mild to moderate ventriculomegaly is reported to increase the risk of abnormal neurodevelopmental outcomes^22^ and to significantly associate with neurocognitive dysfunction^23^. The latter indicates that neuromotor decline following repeated sub-concussion may result from early ventricle enlargement.

Studies demonstrate that the disruption of BBB and CSF-blood barrier may be involved in the development of ventriculomegaly^24^. BBB and CSF-blood barrier play a pivotal role in maintaining CSF protein content and osmotic pressure^25^. Krishnamuthy et al*.* demonstrated in a rat model of intraventricular haemorrhage, the breakdown of BBB resulted in protein and osmotic overload within the ventricles, leading to significant increase in the size of ventricle^26^. Consistent with these reports, in the present study, we found that the rats receiving long-term repeated sub-concussion developed significant BBB disruption, leading to parenchymal perivascular extravasation of blood–borne IgG in the hippocampus and cortex. Furthermore, the correlation analyses showed moderate association between ventriculomegaly and hippocampal BBB disruption, the region adjacent to the lateral ventricle. These data collectively suggest that the dysfunction of BBB induced by the repeated sub-concussive impacts may result in the enlargement of the lateral ventricles, prior to reportable deficits in neuromotor function.

The dysfunction of BBB is increasingly reported following ‘full’ concussion in both humans and animal models^27^. Johnson et al*.* demonstrated substantial BBB disruption at 6–72 h post-injury in a swine model of head rotational acceleration based concussion model, leading to parenchymal extravasation of plasma proteins including IgG^28^. However, the present study is the first to report significant BBB breakdown in repeated sub-concussion. The mechanisms of BBB disruption induced by concussion or sub-concussion are presently unknown. The integrity of BBB can be significantly compromised by exaggerated systematic inflammation. Studies using animal models and in vitro models demonstrate that inflammatory cytokines including IL-6 and TNF-α impair the expression and assembly of BBB tight junction complex, leading to increased BBB permeability^29^. In murine models of dietary-induced inflammation, the inflammation-induced disruption of BBB led to the cerebral leakage of plasma proteins^30,31^. In the current study, the plasma concentrations of pro-inflammatory chemokines and cytokines, including IL-6 and TNF-α, did not show statistically significant differences between the rats receiving sham procedure and repeated sub-concussion. Similarly, anti-inflammatory IL-10 in plasma was comparable between SC and Sham groups. These data suggest that the inflammatory pathways may not be central to the mechanisms of BBB disruption induced by repeated sub-concussive impacts.

Paradoxically, the plasma levels of inflammatory cytokines and chemokines were considerably high even in Sham rats, although the mechanisms are not investigated in the present study. Both Sham and SC rats went through frequent handling as well as forced exercise on rotarod and beamwalk, immediately prior to the sample collection, which may have increased the stress levels in these rats. The latter may be one contributing factor for the exaggerated inflammation observed in this study.

Alterations in intracranial pressure and cerebral blood flow/pressure are also reported to induce increased BBB permeability^32,33^. A study with clinical subjects with idiopathic intracranial hypertension presented substantial BBB dysfunction, resulting in the extravasation of plasma proteins^34^. Significant BBB disruption and parenchymal IgG leakage were also observed in a murine model of dietary-induced hypertension^35^. Indeed, prolonged increase in intracranial pressure is reported in mTBI^36^. Furthermore, severe TBI is often reported to induce systemic hypertension^37^. However, the effects of repeated sub-concussion on intracranial pressure and blood pressure were not measured in this study nor reported previously.

A dysfunctional BBB is demonstrated to significantly promote neuroinflammation and oxidative stress, ultimately leading to neuronal death and neurodegeneration, which may underlie the mechanisms of neurobehavioural decline. In a mouse model of dietary-induced insulin resistance, the disruption of BBB and neuroinflammation preceded neurodegeneration and cognitive decline^30^. Furthermore, in studies using murine models of BBB dysfunction, the protection of BBB integrity with pharmacological and nutraceutical agents completely prevented the disruption of BBB, neuroinflammation and cognitive decline^31,38,39^. In the present study, the rats receiving 2-week repeated sub-concussion showed comparable levels of astrocytosis, microgliosis and neuronal DNA damage to the rats receiving sham procedure, indicating no sign of increased neuroinflammation or oxidative stress. Based on these observations, the neuroinflammatory and oxidative pathways may not be central to the development of neuromotor decline induced by repeated sub-concussion. Alternatively, it can also be speculated that the repeated sub-concussion for only 2 weeks was not sufficient to induce detectable neuroinflammation and oxidative stress. Future studies may consider investigating the effects of repeated sub-concussion for longer periods, e.g. 4 or 8 weeks. Similarly, our present study did not detect any statistically significant changes in the cortical and hippocampal homeostasis of lipids and protein aggregation in the SC rats, potentially due to unsubstantiated insult of 2-week sub-concussion. Nonetheless, non-significant, > 40% reduction was observed in lipid olefinic in corpus callosum and cortex of the SC rats. We have previously applied this spectroscopic approach to identify holistic lipid alterations within white matter during diffuse traumatic brain injury^40^, during diabetes^41^, or during natural ageing^17^. Similarly, this approach has been used to identify altered lipid homeostasis and protein aggregations associated with hippocampal neurodegeneration after ischemic insult^42^. Consistently, in mouse models of Alzheimer’s disease, significant reduction of unsaturated lipid content was detected with FTIR in early stage of the Alzheimer’s pathology development^43^. These data suggest that the reduction of lipid olefinic may be an early sign of neuronal pathologies and neuromotor dysfunction induced by long-term repeated sub-concussion.

No significant changes in neuroinflammation, plasma inflammatory markers or cerebral lipid homeostasis may be a consequent of the protective effects of estrogen. Rats normally have estrouc cycles, which is around 5 days, from 5 weeks of age. Therefore, the rats in the current study may have had 2–3 estrous cycles during the sub-concussion or sham procedure, resulting in transient increase in estrogen which may have exerted protective effects against inflammation.

The present study is the first to demonstrate that repeated sub-concussive impacts for 2 weeks induce significant enlargement of lateral ventricles, preceding neuromotor dysfunction. The data also indicated that the disruption of BBB integrity may be involved in the neuromotor deficits and ventriculomegaly. Our data revealed that the BBB disruption in repeated sub-concussion may occur independent of inflammatory pathways. Whilst the outcomes from rat models may not be directly translatable to clinical, the findings may indicate critical mechanistic pathways for neurobehavioural deficits associated with repeated sub-concussion, and by extension, may offer important therapeutic opportunities.

## Materials and methods

### Animals

Female PVG rats were purchased from Animal Resources Centre (Western Australia) and randomly divided into two groups; a Sham (n = 8) and a sub-concussion (SC) group (n = 8). Rats were acquired at 5–6 weeks of age as to be analogous with clinical findings referenced where neuronal damage was noted in children “heading” soccer balls^44^. Female rats were chosen for continuity with previous studies of concussion and an absence of literature in female animals^18^. All rats were fed standard maintenance chow from Specialty Feeds (Western Australia). All animal procedures in this study were approved by Curtin University Animal Ethics Committee (ARE2019-13).

### Repeated sub-concussion procedure

Sub-concussive impacts were delivered using a customised weight drop apparatus from Northeast Biomedical (MA, USA), as previously described^18,45^. Rats in the sub-concussion group (SC) were anesthetised under 3% gaseous isoflurane prior to repetitive sub-concussive impacts. To simulate repetitive sub-concussive impacts, a 25 g weight was dropped from 1 m height aligned to lambda. This was repeated for a total of 10 impacts and between each impact the site was re-aligned to lambda. Roughly 10–20 s elapsed between each impact. This procedure was repeated 3 days per week for a period of 2 weeks (i.e. Mondays, Wednesdays and Fridays). Sham rats were included in order to eliminate potentially confounding effects of isoflurane anaesthesia, receiving exactly the same anaesthetic regimen as the SC rats without any sub-concussive impacts. Subsequently, rats were placed on a warmed mat prior to regaining consciousness.

### Neuromotor function assessments

At the end of sub-concussion/sham procedure for 2 weeks, rats from both groups were assessed on their neuromotor function within 48 h. This was done utilising a Rotarod and a beam walk test as previously reported^18^. Following an acclimatisation period, the rats were placed on the Rotarod device at a beginning speed of 4 rpm, which was set to accelerate to 40 rpm in 300 s. As the rats fell off, the time elapsed until falling was recorded. The trial was repeated three times and the average was used.

The beam walk test utilised beams of varying width (1, 2 or 3 cm), 30 cm long and 30 cm above the ground. Rats were placed on one side of the beam and observed as they crossed to the other side. The number of foot errors was registered and averaged over 3 attempts on each of the 3 varying beam lengths.

### Sample collection

Following the neuromotor function assessments, the rats were anaesthetised with isoflurane and blood plasma samples were collected into EDTA coated syringes/needles via cardiac puncture. The brain tissue was washed in PBS and divided into hemispheres with an incision on the midline. The right hemisphere was fixed in 4% paraformaldehyde for 24 h and cryoprotected in 20% sucrose for 72 h, before being frozen in dry ice/isopentane for immunofluorescent microscopy analyses. The left hemisphere was frozen in liquid nitrogen immediately for FTIR spectroscopic imaging analyses. The samples were stored at − 80 °C until next use.

### Measurement of lateral ventricle size

The size of lateral ventricles was measured by using the fixed-frozen right hemisphere of the brain. 50 µm thick coronal sections at Bregma 0 mm were prepared by using a rat brain block, and the stereotaxical position was confirmed under a dissection microscope by the anatomy in reference to brain atlas^46^. The sections were stained with hematoxylin and eosin and optical images were captured with Olympus BX-51 microscope at 4 × magnification using Olympus DP70 camera. The size of lateral ventricles was measured by using Zeiss ZEN Blue 3.1 Image Analysis module and expressed as mm^2^.

### Assessment of BBB integrity

BBB permeability was semi-quantitatively measured by a well-established method of cerebral parenchymal IgG leakage utilising an automated machine-learning immunofluorescence confocal microscopy as detailed previously^30,47,48^.

### Plasma inflammatory cytokine measurement

Plasma concentrations of pro- and anti-inflammatory chemokines and cytokines, namely, CXCL1, MCP-1, TNF-α, IL-6, IL-10, IL-12p70, IL-18 and IL-33, were analysed by using a commercial flow cytometry cytokine bead array kit (Biolegend LEGENDplex) as per the manufacturer’s instruction. Briefly, the plasma samples were incubated with supplied pre-mixed beads for 2 h in the dark. Subsequently the beads were incubated with detection antibodies for 1 h and SA-PE for 30 min. The samples were then acquired on BD FACS Canto II by using set up beads. The concentration of each cytokine was extrapolated against a standard curve.

### Detection of neuroinflammation

Measurement of neuroinflammation utilised a previous protocol, described by Mao et al.^9^ with minor modifications. Briefly, 20 µm coronal sections were used from the fixed-frozen right hemisphere. Astrocytosis was detected by rabbit anti-glial fibrillary acidic protein (GFAP, 1:200, Abcam), followed by anti-rabbit IgG Alexa488 (1:200). Microglial activation was determined with goat anti-Iba-1 (1:200, Abcam) followed by anti-goat IgG Alexa555. Similar to the IgG quantitation, immunofluorescent confocal microscopy images were captured with Ultraview Vox microscopy within the entire hippocampal formation and cortex. ZEN Intellesis was trained to recognise astrocytes and microglia for GFAP and Iba-1, respectively, and the mean pixel intensity of GFAP and Iba-1 was measured and expressed for each image area.

### Measurement of neuronal oxidative stress

Neuronal oxidative stress was assessed by using 8-hydroxy deoxyguanosine (8-OHdG) as a marker of oxidised DNA as previously described^9^. The 20 µm fixed-frozen sections were incubated with mouse anti-mouse 8-OHdG (1:500, Abcam) and visualised with anti-mouse Alexa488 (1:200). Similar to the IgG quantitation, confocal images were captures with Ultraview Vox microscopy in the hippocampal formation and cortex. The mean pixel intensity of 8-OHdG staining was measured with Volocity and expressed for each image area.

### Spectroscopic analyses of cerebral biochemical changes

Fourier transform infrared spectroscopy (FTIR) was performed on 10 µm-thick sections of brain tissue, from sham operated and SC animals (*n* = 4 each group) as described previously^41^. Tissue sections were cut using a cryo-microtome at − 16 °C. The sections were melted onto 1 mm thick CaF_2_ optical windows (Crystran) and air-dried before analysis with FTIR. The FTIR spectroscopic images were collected using a Nicolet iN 10MX FTIR microscope, equipped with an 8 × 2 (25 µm) pixel liquid nitrogen cooled linear array detector. Spectra were collected at 8 cm^−1^ spectral resolution with 8 co-added scans, with a background spectrum collected using the same parameters from a blank CaF_2_ substrate.

False-colour functional group images were generated from the FTIR spectroscopic data using Cytospec v2.00.03 and OPUS v7.0. Spectra were vector normalised to the amide I band (1600–1700 cm^−1^) to minimise data variations due to differences in tissue section thickness. Average spectra were calculated from regions of interest that correspond to the hippocampal CA1 pyramidal neuron cell layer, the corpus callosum, and the cortex, as previously described^17,40,41^. For spectra from each region of interest, the integrated area under the curve was calculated for lipid esters [ν(C=O), 1760–1715 cm^−1^], lipid methylene groups [ν_s_(CH_2_), 2865–2840 cm^−1^], and lipid unsaturation [ν(= C–H), 3025–3000 cm^−1^]. Second-derivative spectra were also calculated (9 smoothing point Savitzky–Golay function) and the second-derivative intensity measured at 1625 cm^−1^, as a marker of aggregated β-sheet proteins.

### Statistical analyses

Data normality was assessed with D’Agostino–Pearson omnibus normality test. The normally distributed data are expressed as mean ± standard error of mean (SEM) while the data that were not normally distributed are expressed as median ± 95% confidence interval. In order to assess the statistical difference between Sham and SC groups, the data that were normally distributed were analysed with unpaired two-tailed t-test, whilst the data that failed to pass the normality test were analysed with non-parametric Mann–Whitney test (GraphPad Prism). The associations were assessed with correlation coefficient analyses.

### Ethical approval

The authors confirm that all methods were carried out in compliance with the ARRIVE guideline and in accordance with relevant guidelines and regulations in the method section of the manuscript.

## Supplementary Information

Supplementary Figures.

## Data Availability

All data is presented within the manuscript.
