# Supplementary material for: Blood–brain barrier disruption and ventricular enlargement are the earliest neuropathological changes in rats with repeated sub-concussive impacts over 2 weeks
Source: Sci Rep. 2021 Apr 29;11:9261. doi: 10.1038/s41598-021-88854-9 (PMC8084989; doi:10.1038/s41598-021-88854-9)
Supplement: Supplementary file 1 — Supplementary Figures. [file 41598_2021_88854_MOESM1_ESM.docx]

**Blood-brain barrier disruption and ventricular enlargement are the earliest neuropathological changes in rats with repeated sub-concussive impacts over 2 weeks**

Bailey Hiles-Murison, Andrew P Lavender, Mark J Hackett, Joshua Armstrong, Michael Nesbit, Samuel Rawlings, Terry McGonigle, Andrew Warnock, Virginie Lam, John CL Mamo, Melinda Fitzgerald, Ryu Takechi


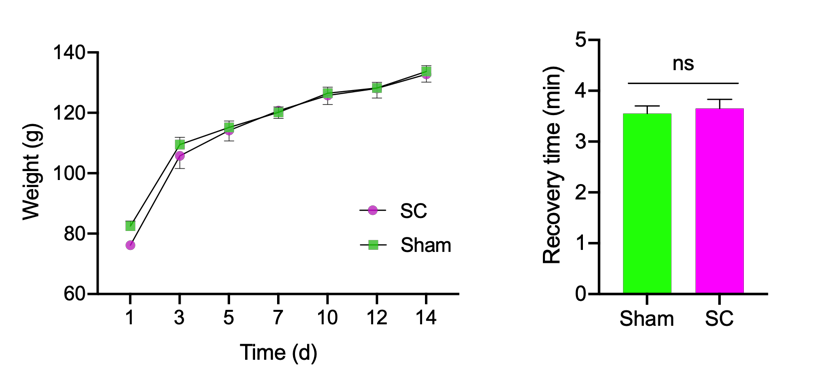


***Supplementary Fig S1. Weights and post procedure recovery time***

*The weight of rats receiving repeated sub-concussion (SC) or sham procedure for 2 weeks were recorded every 2-3 days and presented as mean±SEM. The time took for recovering from the anaesthesia after each SC or Sham procedure was also recorded and presented. Statistical significance was assessed with 2 tailed t-test (p<0.05, n=8).*


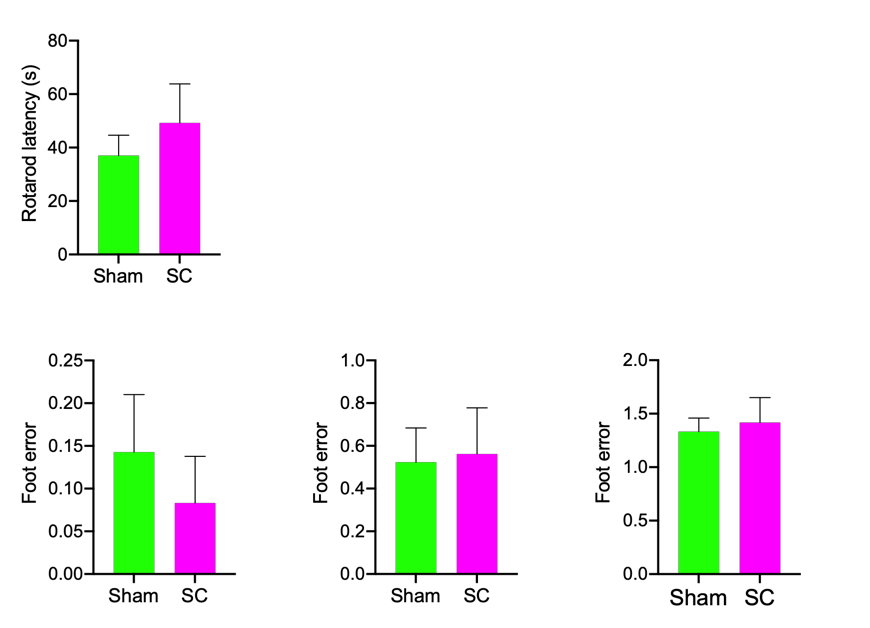


***Supplementary Fig S2. Raw data for neuromotor function tests***

*Neuromotor performance in rats receiving repeated sub-concussion (SC) or sham procedure for 2 weeks were assessed with rotarod and beamwalk tests using 3, 2, and 1 cm. The latency of rats stayed on the rotating rod of rotarod test is presented (mean±SEM). The number of foot errors on 3, 2, and 1 cm beamwalk also shown. Statistical significance was assessed with 2 tailed t-test (p<0.05, n=8).*
